# Triterpenoids and Polysaccharides from *Ganoderma lucidum* Improve the Histomorphology and Function of Testes in Middle-Aged Male Mice by Alleviating Oxidative Stress and Cellular Apoptosis

**DOI:** 10.3390/nu14224733

**Published:** 2022-11-09

**Authors:** Yanhong Li, Wei Liang, Yunlin Han, Wenjie Zhao, Siyuan Wang, Chuan Qin

**Affiliations:** 1 Institute of Medical Laboratory Animal Science, Chinese Academy of Medical Sciences (CAMS) & Comparative Medicine Centre, Peking Union Medical Collage (PUMC), Beijing 100021, China; 2 NHC Key Laboratory of Human Diseases Comparative Medicine, the Institute of Laboratory Animal Sciences, CAMS&PUMC, Beijing 100021, China; 3 Beijing Key Laboratory for Animal Models of Emerging and Remerging Infectious Diseases, the Institute of Laboratory Animal Sciences, CAMS&PUMC, Beijing 100021, China; 4 National Human Diseases Animal Model Resource Center, the Institute of Laboratory Animal Sciences, CAMS&PUMC, Beijing 100021, China

**Keywords:** *Ganoderma* lucidum, middle-aged male mice, testis, mitochondria, reactive oxygen species, apoptosis

## Abstract

Aging is an inevitable physiological process accompanied by a decline in body physiology, including male fertility. A preparation from *Ganoderma lucidum* (GL) containing triterpenes and polysaccharides has been shown to have anti-aging properties. In the current study, the effects of GL on mating ability, testosterone secretion, and testicular structure and function were observed in middle-aged male mice. The GL preparation was administered orally to mice for 2 to 5 months, and then behavioral, serological, and histopathological examinations were performed. Results showed that in the GL group of mice, the mating latency was shortened, the number of pursuits within 20 min was increased, and the mating success rate was higher compared to control mice. Additionally, the levels of serum testosterone, cell proliferation (Ki67), and sperm-specific lactate dehydrogenase (LDH)-C4 were increased, while the levels of senescence-related protein p16 and cellular apoptosis were decreased in GL mice. Testicular spermatogenic cells and sperm and stromal cells were reduced and exhibited structural disorder in 11- and 14-month-old control mice, while these changes were improved compared to age-matched mice receiving the GL preparation. Furthermore, the levels of reactive oxygen species (ROS), malondialdehyde (MDA), and the pro-apoptotic protein Bax were decreased, while the anti-apoptotic protein Bcl-2 was increased in GL mice. Finally, the mitochondrial structure was relatively complete in GL mice compared to controls. Therefore, GL has the potential to improve testicular structure and function in middle-aged male mice by alleviating oxidative stress, maintaining mitochondrial homeostasis, and reducing cellular apoptosis.

## 1. Introduction

Aging is always accompanied by a general decline in body physiology, leading to increased age-related disorders, including cardiovascular, neurodegenerative, and inflammatory diseases. It is also an inevitable physiological process of organisms over time [1]. It has been reported that an imbalance between the increased production of reactive oxygen species (ROS) and shortages in antioxidant scavenging systems with increased age could cause oxidative stress and cellular damage [2]. The reproductive capacity of both males and females declines with age. In males, the effects of aging on the reproductive system include morphological and histological changes in the testes [3]. Reproductive organ aging occurs in humans at the age of 50, which is equivalent to approximately 14 months old in mice [4]. Men older than 50 years produce 30% less sperm in a day, and both sperm motility and morphology are negatively correlated with age [3]. In mice, age-related changes occur during their middle-aged phase (10–15 months), but failure of reproductive organ function occurs primarily at the end of this phage (15 months) [5].

The pharmacological function of *Ganoderma lucidum* (GL) was recorded earlier than 100 BC (Before Christ) in Shen Nong’s *Materia Medica*, where it was described to promote health, increase energy and vitality, and prolong life [6]. Two primary active components that contribute to its anti-aging properties include GL polysaccharides and triterpenes [1].

GL polysaccharides are bound to protein and have an average molecular weight of 1013 kDa, and the main monosaccharides include glucose, galactose, and mannose in the respective proportions of 1:1.28:4.91 [7]. Importantly, oxidative stress is associated with various human diseases and the aging process, and it has been reported that GL polysaccharides can improve aged-related oxidative stress and injury in mouse liver, brain, and spleen [7,8]. GL polysaccharides also have a protective effect on testicular torsion/detorsion-induced ischemia-reperfusion injury and toxic damage in animal experiments, mainly due to their antioxidant properties [9,10]. In addition, they have been shown to have protective effects against testicular damage caused by physical or chemical factors, and function by reducing apoptosis and oxidative stress, as well as regulating sex hormone production [9,10,11].

GL triterpenes are highly oxidized derivatives of lanostane with complex structures and high lipophilicity [12]. GL triterpenes have been shown to exhibit antioxidant characteristics in vitro by scavenging free radicals from cells [13], increasing antioxidant enzymes, and diminishing radiation-induced DNA oxidative injury and apoptosis in mice splenocytes [14]. They can also protect against oxidative-stress-induced protein and lipid peroxidation in liver and brain tissues of aged mice [15,16,17]. GL triterpenes, especially the total triterpene products, have potential anti-aging effects mediated by their antioxidant properties [1]. An ethanol extract of GL has been shown to increase the activities of tricarboxylic acid cycle dehydrogenases and mitochondria electron transport chain complexes, resist the reduced antioxidant capacity in aging brain and heart tissues, and reduce cell damage [1,11,17]. However, there have not been any reports on the effect of GL polysaccharides and triterpenes on testicular tissue aging.

The current study focuses on GL preparations, with polysaccharides and triterpenes as the main components, and their effect on testis morphology and function of middle-aged (9- to 14-month-old) mice. The results from this study can provide the theoretical basis and instructive significance for the clinical use of GL in the prevention of male reproductive system aging.

## 2. Materials and Methods

### 2.1. Animals

A total of 45 healthy 9-month-old male specific pathogen-free (SPF) C57BL/6J mice were purchased from Beijing Huafukang Biotechnology Co. Ltd. (SCXK, Beijing, China; 2019-0008). Nine mice were used for baseline data collection and analysis (9-month time point), and the remaining mice were equally divided into two groups (*n* = 18/group): control group (normal chow) and model group (GL-modified chow). Six mice from each group were selected for mating experiments (after 2.5 months on specific diet), and the remaining mice (*n* = 12/group) were used to observe age-related the pathological changes (*n* = 6/group at each time point: 11- and 14-month time points). Healthy 3-month-old female SPF C57BL/6J mice (after 2.5 months on specific feeding routine under the same conditions; *n* = 12) were used in mating experiments at a ratio of 1:1. Animals were kept in the SPF Animal Room of the Institute of Medical Experimental Animals, Chinese Academy of Medical Sciences (SYXK, Beijing; 2019-0014). Except for mating experiments, mice were housed at a maximum of five per cage and maintained at a constant temperature (22–26 °C) and relative humidity (60–70%), on a 12 h dark/light cycle. Mice had access to food and water ad libitum. The use of animals and related procedures were approved by the Laboratory Animal Use and Management Committee of the Institute of Medical Laboratory Animals, Chinese Academy of Medical Sciences (IACUC Approval Number: QC19022), and experiments were performed in accordance with the International AAALAC Guidelines on the Welfare and Use of Laboratory Animals.

### 2.2. Materials

GL spore powder (GLSP) capsules were used, the ingredients of which include GL fruiting body, hawthorn, and broken spores, and their composition ratio is 3.3:3.3:1. The formation process was as follows: GL fruiting body and hawthorn were mixed at a ratio of 1:1, then hot water processing was performed a total of three times, where 100 °C water was added and the heat of the mixture was maintained for 1 h. The mixed solution was concentrated, dried (75 °C), and crushed to obtain the extract. After breaking the wall of the GL spores, the powder was added to the extract, which was then mixed, granulated, and used to fill capsules. The active ingredients and content of the GLSP capsules included total triterpenes (5.4%) and polysaccharides (4.34%). GL spore oil (GLSO) (100%) was extracted from broken spores by supercritical carbon dioxide extraction, and the active ingredient was primarily *Ganoderma* triterpenoids (28.1%). GLSP capsules and GLSO were provided by MeiShanTang Biotechnology (Shenzhen, China) Co., Ltd. GLSP and GLSO were homogeneously mixed with the standard mouse chow to make GL-modified chow [18]. Purchase of the standard chow and generation of the GL-modified chow was completed by Beijing Huafukang Biotechnology Co., Ltd. (SCXK, Beijing, China; 2019-0008). The goal was to administer GLSO and GLSP to animals through feeding at a dose of 0.6 g/kg body weight/day (according to the product instructions and the dose for healthy adults following body surface area conversion [17,19]). Animals in the control group were provided standard chow with no added ingredients under the same conditions.

### 2.3. Animal Mating Experiment

Middle-aged male mice (11.5 months old) in the model group (GL-modified chow for 2.5 months) and control group (standard chow for 2.5 months) were mated with 5-month-old healthy adult female mice at a ratio of 1:1 (one mating pair per cage). Once the male and female mice were introduced, the time between the introduction and the first occurrence of mating (mating latency) as well as the number of pursuit and mounting events within a single 20 min mating period were observed [20,21]. Subsequently, the mating pairs were housed together for four months. The success rate after mating, the number of fetuses, and the average number of newborn mice per mating pair were observed during this 4-month period. Generally, the spermatogenic cycle is 35 days in male mice [22] and the estrus cycle of female mice is 4–5 days [23].

### 2.4. Measurements of Body Weight and Weight of Testis

The body weight measurements of male mice at 9, 11, and 14 months old were completed in both the model and control groups. After dissection at each time point, the testicular tissues of mice were removed and weighed. The organ coefficient (=weight of testes/body weight × 100%) was calculated.

### 2.5. Detection of Serum Testosterone

Blood samples were collected from the orbital venous plexus of mice at 9, 11, and 14 months old, and centrifuged twice (5424R, Eppendorf) for 20 min at 3000 rpm. Serum samples were collected and stored at −80 °C until analysis. Testosterone was detected in serum samples using a Luminex assay (liquid suspension chip technique; LX-MADKMAG-21K-05, Millipore, Shanghai, China).

### 2.6. Pathologic Analysis

Mice in the model and control groups at 9, 11, and 14 months old were euthanized by cervical dislocation and dissected. Tissue samples were collected and fixed in 10% neutral formalin, dehydrated in an ethanol gradient (70–100%), embedded in paraffin, and sectioned (5 μm slices). Sections were stained with hematoxylin and eosin (H&E) for pathological analysis. For staining, the sections were dewaxed and rehydrated, stained by hematoxylin, differentiated using hydrochloric acid and alcohol, then stained with eosin after bluing in ammonia water. Slices were sealed after dehydration and transparency, and stained sections were observed under a light microscope (BX51, Dongjing, Japan).

### 2.7. Aging-Related Molecular Assays of Tissue

Immunohistochemistry was used for detection of sperm-specific lactate dehydrogenase LDHC (bs-3827R, Beijing, China) and cell senescence protein p16 (sc-1661, Shanghai, China) in testicular tissue [24,25]. Paraffin sections were dewaxed and rehydrated, antigen retrieval was performed using a microwave for 10 min, endogenous peroxidase activity was quenched with hydrogen peroxide, and goat serum was added to block non-specific staining. Next, primary antibodies were added at 4 °C overnight, and corresponding enzyme-labeled secondary antibodies were used. Staining was developed using a colorant. Slices were washed with tap water, counterstained with hematoxylin, dehydrated, cleared with xylene, and covered using neutral glue. The stained sections were observed under light microscopy. For each section, three to five visual fields of view were selected for average optical density analysis of the indicator using Image-pro plus software. The protocol and statistical method were according to the instruction of software, as follows: intensity calibration—std option density; option—image; count/size; IOD/area—save settings; select colors—HSI (0–30; 0–255; 0–255) and save; count; statistics; IOD SUM; irregular drawing; count/size; edit—convert AOI to object—area. The average optical density = IOD SUM/area.

### 2.8. Detection of Cellular Apoptosis and Proliferation

Cellular apoptosis within testicular tissue was detected by Terminal Deoxynucleotidyl Transferase-Mediated Nick End Labeling (TUNEL) (S7101, Shanghai, China) staining [26], which was performed according to the provided instructions. Paraffin sections were dewaxed and rehydrated, and incubated with proteinase K solution (20 μg/mL) at room temperature (RT) for 15 min. Endogenous peroxidase activity was quenched with 2% hydrogen peroxide for 5 min at RT. TdT reaction buffer was added and incubated for 60 min at 37 °C. BSA (2%) solution was added to block nonspecific binding for 30 min at RT, then TdT peroxidase was used for 1 hr at 37 °C. Slices were stained using DAB staining solution at RT, then washed with tap water and covered with coverslips using mounting medium. The sections were observed under a light microscopy. The number of positive cells in each field was also detected using Image-pro plus software. The method was as follows: count/size—segmentation—HSI (0–30; 0–255; 0–255)—save; count/size—manual—select colors—measure; view—statistics—samples.

Additionally, levels of pro-apoptosis protein Bax (ab32503, Cambridge, UK) and anti-apoptosis protein Bcl-2 (ab59348, Cambridge, UK), as well as Ki67 (ab15580, Cambridge, UK; marker of cellular proliferation), were detected in testicular tissue by immunohistochemistry [24,25]. The statistical method of Bax and Bcl-2 was the same as LDHC and p16. The statistical method of Ki67 was the same as TUNEL.

### 2.9. Detection of Oxidative Stress Molecules in Testes

Immunohistochemistry [24,25] was used to detect the levels of reactive oxygen species (ROS) in testicular tissues using an anti-reactive oxygen cluster ROS antibody (nbp2-13245, Littleton, CO, USA). The levels of malondialdehyde (MDA) in testicular tissue homogenates were measured using the MDA detection kit (BC0020, Beijing, China) according to the instructions provided [25]. The testicular tissue and extracting solution were homogenized in an ice bath at a ratio of 1:5, and then centrifuged at 8000× *g*, 4 °C for 10 min, and the supernatant was collected. The supernatant samples and MDA detection working solution were prepared according to the requirements of the kit, mixed well, incubated at 100 °C for 30 min, and then centrifuged at 25 °C, 10,000× *g* for 10 min. The absorbance values of the samples were measured at 450 nm, 532 nm, and 600 nm. MDA content (nmol/g) = (6.45 × (A532 − A600) − 1.29 × A450) × V_total_/(W_sample mass_ × V_sample_/V_extraction_).

### 2.10. Electron Microscopy Examination 

Testicular tissues were harvested immediately after the animals were euthanized. Half of the tissue was fixed in formaldehyde for paraffin sectioning, while the other half was placed in a 2.5% glutaraldehyde phosphate buffer solution for 2% osmium acid fixation and epoxy resin embedding. Ultrathin sections were cut out using an ultrathin microtome. Sections were stained with lead citrate and uranium acetate and observed under a transmission electron microscope (JEM-1400, Tokyo, Japan). 

### 2.11. Statistical Analysis

The images of tissue sections were analyzed using Image-pro plus software (Media cybernetics, Inc. IPP 6.0, Rockville, MD, USA). Data were analyzed using Microsoft Excel (Microsoft Corporation, Redmond, WA, USA) and expressed as mean ± standard deviation (MEAN ± SD). Comparisons between groups were performed using *t*-tests (T.TEST) and a *p*-value < 0.05 was considered statistically significant.

## 3. Results

### 3.1. GL Preparation Improves Sexual Function in Male Mice

**Figure 1:**
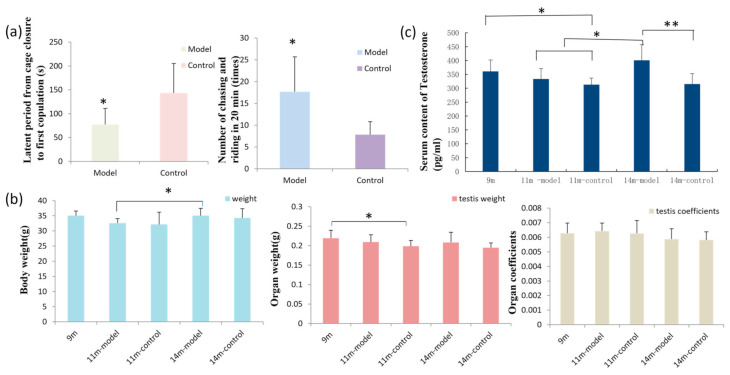
Detection of male physiological indexes in mice. (**a**) The mating latency period from cage closure to first copulation. (**b**) Body weight, testicular weight, and organ coefficient of mice. (**c**) Serum testosterone levels in mice of different ages; *n* = 6–8 in each group; * *p* < 0.05 and ** *p* < 0.01 vs. controls.

Mating experiments on 11.5-month-old mice showed that the mating latency of mice in the model group was shorter (*p* < 0.05), and they pursued the females more times (*p* < 0.05) compared to the control mice within the 20-minute mating exposure. After mating, the rate of successful pregnancy for the first litter was 83.3% (5/6) in the model group and 66.7% (4/6) in the control group (Figure 1a).

Over four months of mating, in the model group the rates of successful pregnancies were 50% (3/6) for the second litter, 33.3% (2/6) for the third litter, and 33.3% (2/6) for the fourth litter. The average number of births per litter was 5 (56/12). Over the same period in the control group, the rates of successful pregnancies were 50% (3/6) for the second litter, 33.3% (2/6) for the third litter, and 0% (0/6) for the fourth litter. The average number of births per litter was 3 (31/9) (Table 1). In conclusion, the mice receiving GL had higher rates of successful mating, more total births, and a higher average of mice per litter compared to the control group.

### 3.2. Results of Body Weight, Organ Weight, and Organ Coefficient

The body weights of mice at different ages were measured, and 14-month-old model mice were found to weigh more than 11-month-old model mice (*p* < 0.05). There was no significant difference in testicular weight of age-matched mice between the model and control groups. However, testicular weights were significantly decreased in the 11-month-old control group compared to 9-month-old mice (*p* < 0.05), but there was no significant difference among the other groups. Additionally, there was no significant difference in the organ coefficient among the groups (Figure 1b).

### 3.3. GL Preparation Increases Serum Testosterone Levels in Middle-Aged Male Mice 

Serum levels of testosterone in the control male mice decreased with age, and 9-month-old mice had higher levels compared to 11-month-old control mice (*p* < 0.05). However, testosterone levels were increased in older mice receiving GL. Importantly, 14-month-old mice had higher testosterone levels than 11-month-old model and control mice (*p* < 0.05) and 14-month-old control mice (*p* < 0.01) (Figure 1c). The coefficients of variation (CV = SD/MEAN × 100%) of the measured values in 9-month-old mice, 11-, and 14-month-old model and control mice were 11.39%, 11.43%, 7.57%, 14.06%, and 11.95%, respectively.

### 3.4. Pathological Morphology and Electron Microscopy

**Figure 2:**
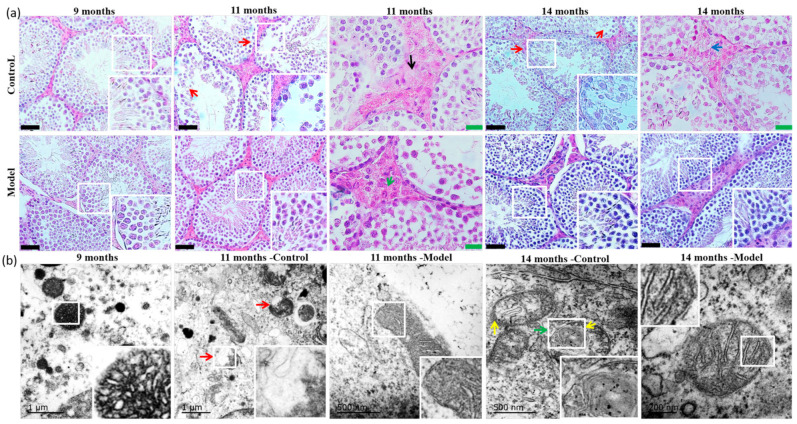
Morphological and ultrastructural analysis of testes in mice at different ages. (**a**) Pathological H&E staining results in testes of mice aged 9, 11, and 14 months (*n* = 6/group). The red arrows show the spermatogenic cells arranged disordered and decreased, and sperm decreased. Black arrow shows the degeneration of Leydig cells. Green arrow shows normal Leydig cells, and blue arrow shows vacuolation of Leydig cells. Black scale bar = 50 μm. Green scale bar = 20 μm. (**b**) Electron microscopic results of testicular tissue of mice aged 9, 11, and 14 months (*n* = 2/group). Red arrows show the destruction of mitochondrial bilayer membrane and the disappearance of crista. Green arrow shows that the mitochondrial bilayer membrane was incomplete, and yellow arrow shows that the cristae were unclear.

In the pathomorphological study, sections of testicular tissue (two per animal) from each group were analyzed, and five to six randomly selected fields of view per each section were evaluated. In 9-month-old mice, spermatogenic cells in seminiferous tubules arranged relatively neatly and occasionally disordered. Leydig cells in the testis had clear morphology, and sperm decreased slightly in some seminiferous tubules compared to the sperm quantity in normal seminiferous tubules. In 11- and 14-month-old control mice, spermatogenic cells and sperm were decreased, along with reduced Leydig cells that showed vacuolar degeneration and lipid deposition. The tissue morphology and structure of the testes in model mice were also disordered and damaged to some extent, but spermatogenic cells in most of seminiferous tubules could be seen at every stage, and the arrangement was more organized compared to age-matched controls (Figure 2a, Table 2).

Most of the testicular mitochondria in 9-month-old mice had intact double-layer membranes, mitochondrial cristae that were short, irregularly and closely arranged, and localized vacuoles. In 11-month-old control mice, most of the testicular mitochondria had abnormal structures, the double-layered membranes were damaged, and the mitochondrial cristae were not apparent. The testicular mitochondria in the 11-month-old model group mice also displayed mitochondrial membrane damage and a lack of cristae, but to a lesser extent. In the testis of 14-month-old control mice, most of the mitochondrial bilayer membranes were incomplete, the cristae were dissolved, and the structure was unclear. However, the mitochondrial changes in the 14-month-old model mice were relatively mild, and the cristae clearly arranged (Figure 2b).

### 3.5. Detection of LDH-C4 and p16 in Testis Tissue

**Figure 3:**
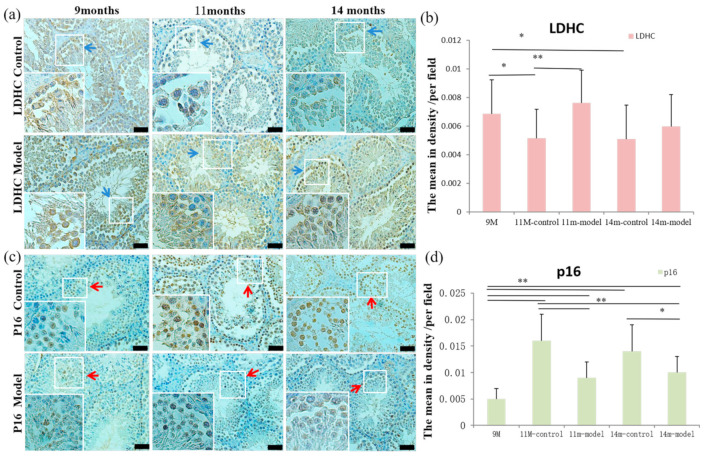
Detection of LDH-C4 and p16 protein expression in testicular tissue of mice. (**a**) Expression levels of sperm-specific enzyme LDH-C4 in testis of mice aged 9, 11, and 14 months. Blue arrows show the positive expression (brownish yellow) of the target protein. (**b**) Statistical results of LDH-C4 expression. (**c**) Levels of senescence protein p16 in testicular tissue of mice aged 9, 11, and 14 months. Red arrows show the positive expression (brownish yellow) of p16 protein. (**d**). Statistical results of p16 expression. Scale bar = 50 μm, *n* = 6 in each group; * *p* < 0.05 and ** *p* < 0.01 vs. controls.

The testicular sperm-specific enzyme LDH-C4 was mainly expressed in spermatocytes, spermatids, and spermatozoa, and the average optical density per field in 11- and 14-month-old control mice was lower compared to 9-month-old mice (*p* < 0.05). Additionally, 11-month-old control mice also had lower LDH-C4 values than 11-month-old model mice (*p* < 0.01) (Figure 3a,b). Senescence-related protein p16 was mainly expressed in the nucleus, and the average optical density per field in testicular tissue was higher in 11- and 14-month-old model and control mice compared to 9-month-old mice (*p* < 0.01) and was lower in 11- and 14-month-old model mice compared to age-matched controls (*p* < 0.05; *p* < 0.01) (Figure 3c,d).

### 3.6. Detection of Cell Proliferation and Apoptosis in Testicular Tissue

**Figure 4:**
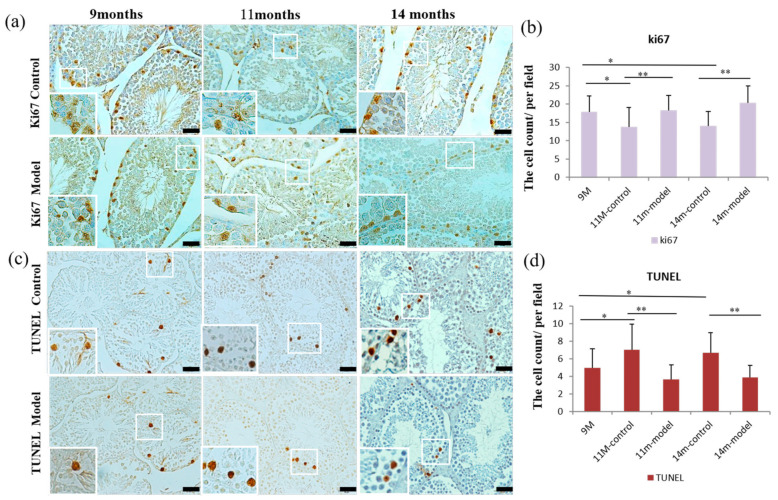
Levels of proliferation and apoptosis in testicular tissue of mice. (**a**) Cell proliferation (Ki67) staining in testes of mice aged 9, 11, and 14 months. (**c**) Apoptosis staining (TUNEL) in testicular tissue of mice aged 9, 11, and 14 months. (**b**,**d**) Statistical results of cell proliferation and apoptosis. Scale bar = 50 μm and *n* = 6 in each group; * *p* < 0.05 and ** *p* < 0.01 vs. controls.

Results from the cellular proliferation analysis in testicular tissue showed that the nucleoprotein Ki67 was expressed in the nucleus of proliferative cells, and the number of positive cells per field of view in 11- and 14-month-old control mice was lower compared to 9-month-old mice (*p* < 0.05), and was also lower compared to age-matched model mice (*p* < 0.01) (Figure 4a,b).

**Figure 5:**
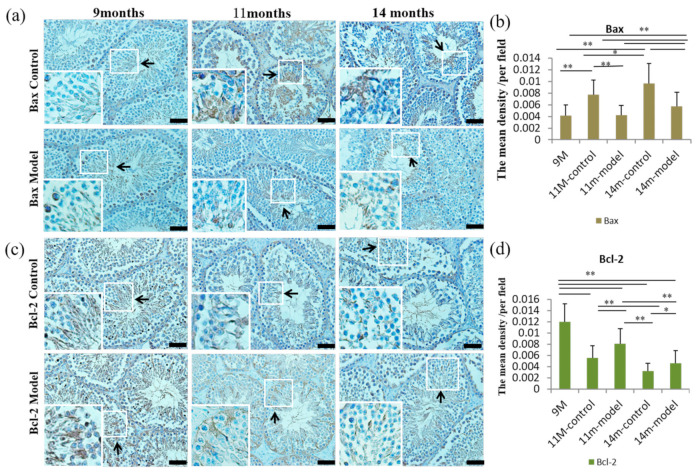
Levels of apoptosis-related proteins Bax and Bcl-2 in testicular tissue of mice. (**a**) Detection of pro-apoptotic protein Bax in testes of 9-, 11-, and 14-month-old mice. The positive expression of Bax protein showed brown-yellow (black arrows). (**c**) Detection of anti-apoptotic protein Bcl-2 in testes of 9-, 11-, and 14-month-old mice. The positive expression of Bax protein showed brown-yellow (black arrows). (**b**,**d**) Statistical results of apoptosis-related protein expression. Scale bar = 50 μm and *n* = 6 in each group; * *p* < 0.05 and ** *p* < 0.01 vs. controls.

Following the detection of apoptosis, the number of apoptotic cells in 11- and 14-month-old control mice was increased compared to the 9-month-old mice (*p* < 0.05) and age-matched model mice (*p* < 0.01) (Figure 4c,d). Further, the average optical density per field of view of the pro-apoptotic protein Bax in 11- and 14-month-old model mice was lower compared to age-matched controls (*p* < 0.01). However, levels of Bax were higher in 11-month control as well as 14-month model and control mice compared to 9-month-old mice (*p* < 0.01). In 14-month-old model and control mice, Bax levels were increased compared to 11-month-old model (*p* < 0.01) and control mice (*p* < 0.05) (Figure 5a,b). Levels of the anti-apoptotic protein Bcl-2 were higher in 11- and 14-month-old model mice compared to age-matched controls (*p* < 0.01; *p* < 0.05). Bcl-2 was highest in 9-month-old mice compared to all other groups (*p* < 0.01), and 14-month-old control and model mice had higher levels of Bcl-2 than 11-month-old control and model mice, respectively (*p* < 0.01) (Figure 5c,d).

### 3.7. ROS and MDA Levels in Testicular Tissue

**Figure 6:**
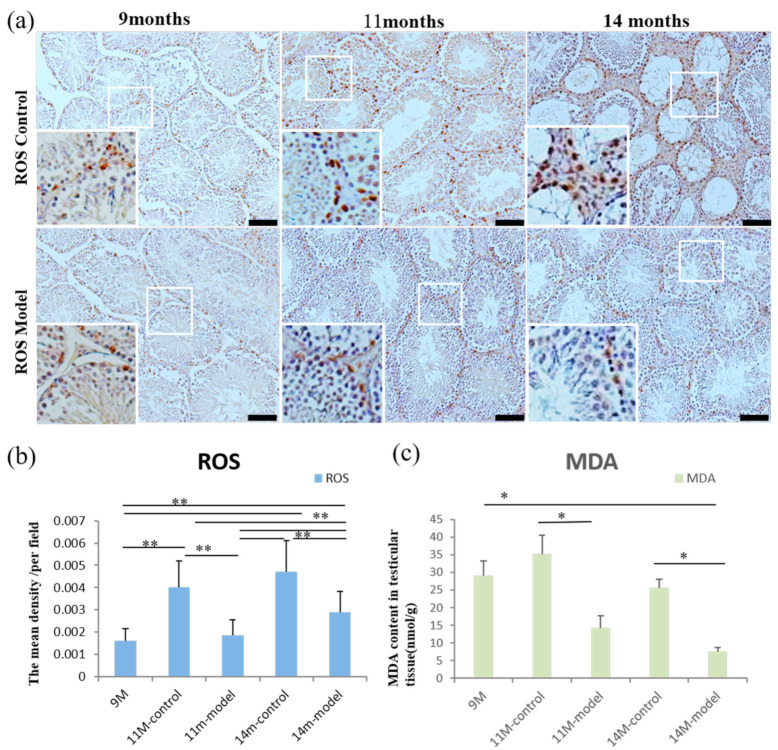
Detection of ROS and MDA in testicular tissue. (**a**) Expression of ROS in testes of 9-, 11-, and 14-month-old mice. (**b**) Statistical analysis of ROS levels in testes of mice. Scale bar = 100 μm and *n* = 6 in each group. (**c**) MDA content in testicular tissue homogenates. * *p* < 0.05 and ** *p* <0.01 vs. controls.

The average optical density per field of view of ROS in the testicular tissue of 11- and 14-month-old control mice was significantly increased compared to 11- and 14-month-old model mice as well as 9-month-old mice (*p* < 0.01). ROS levels in 14-month-old model mice were higher compared to 11-month-old model and 9-month-old mice (*p* < 0.01) (Figure 6a,b). Testicular tissue homogenate was used to detect levels of oxidative stress product MDA, and MDA levels were lower in the 11- and 14-month-old model mice compared to the age-matched controls (*p* < 0.05) (Figure 6c).

## 4. Discussion

This study was the first to show that GL preparations containing triterpenes and polysaccharides may significantly improve the morphology and function of testicular tissue in middle-aged male mice. The effect may be mediated through the reduction in ROS caused by aging, while it ultimately reduces oxidative stress, helps to maintain mitochondrial homeostasis, and reduces apoptosis. This study provides a potential therapeutic strategy for the clinical prevention of male reproductive system aging. Limitations of this study included the oral administration of the preparation, which has some impact on dose assessment, and the small sample sizes of experimental animals.

In the current study, the oral administration of the preparation was selected mainly because long-term gavage inevitably causes damage to the esophagus and stomach of animals. The chow was given quantitatively according to the daily food intake of the mice, which was consistent with the controls. This method of administration has also been adopted in our previous study [18]. It proved that there was not much waste, the error caused by which was small, and the research results were not affected. In addition, GL is safe and has less adverse effects [12], and its antioxidant function is dose-dependent [7,17]. Therefore, considering the heterogeneity of daily intake, the dosage based on the recommended dose was increased to better evaluate the effects of GL on the morphology and function of testis in middle-aged mice.

Firstly, the morphology and function of the testicular tissue was compared between the model and control groups through behavioral, histopathological, and molecular assays. Then, a possible mechanism was considered. Sperm-specific LDH-C4 is only present in the testes and sperm of mammals and birds and plays an important role in sperm energy metabolism and capacitation [27]. LDH-C4 is present in the cytoplasm of testicular cells from spermatocytes to spermatids, primarily in mitochondria and cytoplasmic membranes [28]. It has been reported that a gene disorder of LDH-C4 in male mice can cause sperm motility disorders and infertility [29]. In the current study, LDH-C4 expression in 11-month-old model mice was found to be higher than that in age-matched control mice, and expression in 11- and 14-month-old control mice was lower compared to 9-month-old mice. These data indicate that administration of the GL preparation increases the expression of LDH-C4 in the testes of middle-aged male mice. When analyzing the microstructure of the testes, the morphology and structure of the mitochondria in model mice were relatively complete, further revealing that GL may play a role in maintaining the morphology and function of testicular tissue and sperm.

Next, through mating experiments, the mating latency of male mice in the model group was found to be shortened, the number of pursuits per unit time increased, and the mating success rate was improved, suggesting that GL played a role in improving testicular function and sperm activity. In addition, testosterone is the main anabolic hormone in men and a key factor in maintaining normal reproductive and sexual function, and serum testosterone levels decrease with age [30]. In the current study, serum testosterone levels in the 14-month-old model mice were significantly increased compared to age-matched controls, as well as the 11-month-old model and control mice, which indicates that GL can promote the secretion of testosterone. Since testosterone is secreted from the testes [31], this further indicates that GL may promote the increase in testosterone secretion through the maintenance of testicular morphology and function.

The p16 gene is not only a tumor suppressor gene but is also considered to be a dominant gene of cellular senescence and plays an important role in its initiation and maintenance [32]. The expression of p16 increases significantly with age in most tissues of mice [32]. In addition, studies have shown that it is overexpressed in senescent fibroblasts with oxidative stress, DNA damage, and altered chromatin structure [32]. In the current study, p16 expression in the testicular tissue of 11- and 14-month-old control mice was higher compared to age-matched model mice as well as 9-month-old mice. The reduced expression of p16 in the model group further verified that the structure and morphological function of the testes in this group were better than those in the control group.

There is accumulating evidence to show that oxidative stress plays an important role in the pathogenesis of aging, and it mainly occurs during an imbalance between antioxidant defense and increased ROS [7]. ROS is an important mediator for signal transduction and plays a role in sperm capacitation and the acrosome reaction, which regulates the association between sperm and oocyte [33,34,35]. However, high levels of ROS can cause a variety of cellular and DNA damage [36,37,38,39,40,41]. Mitochondrial DNA has no histone wrapping and repair mechanism, so it is more vulnerable to damage than nuclear DNA [42]. Mitochondrial damage can in turn lead to increased ROS production and oxidative stress and decreased ATP levels [43,44,45]. These injuries can cause mitochondrial aberration and activation of the cell death pathway [42,46]. Excessive accumulation of ROS leads to an increase in oxidative stress, and MDA levels are usually evaluated as a final product of lipid peroxidation [7]. Studies have shown that an increase in ROS is negatively correlated with sperm function [47]. The imbalance between the antioxidant defense mechanism and ROS production during aging process eventually leads to DNA, lipid, and protein damage [48]. Therefore, as men grow older, increased oxidative stress is related to decreased sperm motility and increased sperm DNA fragmentation [5]. The results of the current study showed that the levels of ROS and MDA in the testes of the model group were decreased compared to age-matched control mice, which indicated that GL could reduce the oxidative stress caused by excessive production of ROS and MDA in testicular tissue of middle-aged mice.

Oxidative stress leads to increased lipid peroxidation, DNA damage, and apoptosis, and can ultimately result in the loss of sperm motility [1,49,50,51,52,53]. Although apoptosis is a necessary condition for spermatogenesis under normal circumstances, with an increase in age, the balance between apoptosis in different types of germ cells and spermatogonia proliferation is disturbed [54]. In the spermatogonia of the elderly, the expression of Ki67 (marker of cellular proliferation) is decreased and the rates of spermatocyte apoptosis are increased, which may explain the mechanism of germ cell reduction caused by aging [55]. The current results revealed that rates of apoptosis in the testes of middle-aged mice were significantly higher, while cellular proliferation was significantly lower, compared to 9-month-old mice. A study on the histology and ultrastructure of the testis of elderly men showed that the proliferation of germ cells decreases while apoptosis increases [56], which was consistent with our experimental results. Compared with age-matched control mice, cellular apoptosis was decreased and cellular proliferation was increased in 11- and 14-month-old model mice. These results further indicate that GL may improve mitochondrial structure and reduce apoptosis by lowering oxidative stress, ultimately improving the state and maintaining the function of testicular germ cells.

Apoptosis-related proteins Bcl-2 and Bax participate in the mitochondrial-mediated apoptosis pathway. Bcl-2, an anti-apoptosis protein, prevents caspase activation by preventing cytochrome c and apoptosis-inducing factor from being released from mitochondria to cytoplasm, or isolating caspase [57]. The pro-apoptosis protein Bax usually exists in cytoplasm and mitochondria. During apoptosis, cytoplasmic Bax in the form of a monomer moves to the mitochondria and forms a dimer. That is, during the process of apoptosis, cytoplasmic Bax undergoes conformational changes, showing homogeneous dimerization/translocation to the mitochondrial membrane, which may lead to apoptosis by causing mitochondrial dysfunction [57,58]. The current study further showed that the expression of anti-apoptosis protein Bcl-2 was increased in 11- and 14-month-old model mice compared to age-matched control mice. It can be speculated that increasing the expression of anti-apoptotic protein Bcl-2 and inhibiting the expression of pro-apoptotic protein Bax helps to maintain mitochondrial morphology and function. Therefore, reducing apoptosis may also be one of the mechanisms whereby GL improves testicular morphology and function.

## 5. Conclusions

In summary, GL preparation was shown to protect against age-related changes to the structure, morphology, and function of testicular tissue in middle-aged male mice. These effects may be mediated by the inhibition of ROS and MDA overproduction caused by age, with additional inhibition of oxidative stress, promotion of Bcl-2 expression, and inhibition of Bax expression. Overall, this serves to maintain mitochondrial structure and function and reduce apoptosis. However, there may be other mechanisms at play, such as immune regulation, which were not within the scope of this study. Additional experiments are required to expand the sample sizes of animal use and obtain a comprehensive and in-depth analysis of other molecular mechanisms related to male reproductive system protection by GL.

## Figures and Tables

**Table 1:** The number of fetuses and litter size of the two groups after mating (*n* = 6).

Group	Model	Control
Birth ratio and litter size of the first litter	5/6 (83.3%); 23	4/6 (66.7%); 17
Birth ratio and litter size of the second litter	3/6 (50%); 19	3/6 (50%); 10
Birth ratio and litter size of the third litter	2/6 (33.3%); 10	2/6 (33.3%); 4
Birth ratio and litter size of the fourth litter	2/6 (33.3%); 10	0; 0
Average litter size per litter	4.67	3.4

**Table 2:** Histopathological changes of testicular tissue.

Group	Disordered Arrangement of Spermatogenic Cells	Spermatogenic Cell and Spermatozoa Decrease	Leydig Cell Reduction, Degeneration, and Lipid Deposition
9-month	18.3%	15.4%; 16.9%	14.3%; --; --
11-month control	50.7%	47.2%; 51.4%	31.4%; 17.1%; 8.6%
11-month model	38.5%	30.5%; 31.9%	24.3%; 14.3%; 5.7%
14-month control	65.7%	60%; 70%	41.4%; 31.4%; 18.6%
14-month model	47.1%	42.8%; 47.1%	30%; 20%; 12.9%

*n* = 6 mice/group, *n* = 12 testicular tissue/group, n’ = 60–72 fields/group. The value in the table = number of visual fields with corresponding lesions/total number of visual fields × 100%; “--”: Normal.

## Data Availability

Not applicable.
